# Deregulation of Ca^2+^-Signaling Systems in White Adipocytes, Manifested as the Loss of Rhythmic Activity, Underlies the Development of Multiple Hormonal Resistance at Obesity and Type 2 Diabetes

**DOI:** 10.3390/ijms22105109

**Published:** 2021-05-12

**Authors:** Egor A. Turovsky, Maria V. Turovskaya, Vladimir V. Dynnik

**Affiliations:** 1 Institute of Cell Biophysics of the Russian Academy of Sciences, Federal Research Center, Pushchino Scientific Center for Biological Research of the Russian Academy of Sciences, 142290 Pushchino, Russia; turovsky.84@mail.ru (E.A.T.); m_turovskaya@mail.ru (M.V.T.); 2 Institute of Theoretical and Experimental Biophysics, Russian Academy of Sciences, 142290 Pushchino, Russia

**Keywords:** murine white adipocytes, G proteins interplay, feedback control of Ca^2+^ signaling systems, Ca^2+^ oscillations and triggering phenomena, NO and protein kinase G, loss of rhythmicity and general hormonal resistance to obesity

## Abstract

Various types of cells demonstrate ubiquitous rhythmicity registered as simple and complex Ca^2+^-oscillations, spikes, waves, and triggering phenomena mediated by G-protein and tyrosine kinase coupled receptors. Phospholipase C/IP_3_-receptors (PLC/IP_3_R) and endothelial NO-synthase/Ryanodine receptors (NOS/RyR)–dependent Ca^2+^ signaling systems, organized as multivariate positive feedback generators (PLC-G and NOS-G), underlie this rhythmicity. Loss of rhythmicity at obesity may indicate deregulation of these signaling systems. To issue the impact of cell size, receptors’ interplay, and obesity on the regulation of PLC-G and NOS-G, we applied fluorescent microscopy, immunochemical staining, and inhibitory analysis using cultured adipocytes of epididumal white adipose tissue of mice. Acetylcholine, norepinephrine, atrial natriuretic peptide, bradykinin, cholecystokinin, angiotensin II, and insulin evoked complex [Ca^2+^]_i_ responses in adipocytes, implicating NOS-G or PLC-G. At low sub-threshold concentrations, acetylcholine and norepinephrine or acetylcholine and peptide hormones (in paired combinations) recruited NOS-G, based on G proteins subunits interplay and signaling amplification. Rhythmicity was cell size- dependent and disappeared in hypertrophied cells filled with lipids. Contrary to control cells, adipocytes of obese hyperglycemic and hypertensive mice, growing on glucose, did not accumulate lipids and demonstrated hormonal resistance being non responsive to any hormone applied. Preincubation of preadipocytes with palmitoyl-L-carnitine (100 nM) provided accumulation of lipids, increased expression and clustering of IP_3_R and RyR proteins, and partially restored hormonal sensitivity and rhythmicity (5–15% vs. 30–80% in control cells), while adipocytes of diabetic mice were not responsive at all. Here, we presented a detailed kinetic model of NOS-G and discussed its control. Collectively, we may suggest that universal mechanisms underlie loss of rhythmicity, Ca^2+^-signaling systems deregulation, and development of general hormonal resistance to obesity.

## 1. Introduction

Intracellular calcium-signaling machinery is known to be implicated in the regulation of diverse cellular functions in many types of cells and tissues. Numerous Ca^2+^-signatures, evoked by various hormones acting via G-protein and tyrosine kinase coupled receptors (GPCR and RTK), are decoded to provide specific responses in the cells. However, the mechanisms of decoding remain an open question, in spite of some attempts to apply specific mechanisms based on target proteins’ Ca^2+^-sensitivity shapes, amplitude and frequency filtering, etc., [1,2,3,4]. The temporal patterns of calcium response to hormonal signal in various types of cells may include a slow or steep rise of [Ca^2+^]_i_ and Ca^2+^-spikes [5,6], simple and complex Ca^2^ oscillations [7,8,9,10,11,12,13], triggering phenomena (switching of the system between two states with different [Ca^2+^]_i_ levels) [8], or intra-and intercellular Ca^2+^-waves [10,14,15]. At this time, such complex Ca^2+^-responses were registered in most type of cells, including non-excitable and excitable cells, which indicates the operation of positive feedback loops (PFL) in cellular Ca^2+^-signaling systems [16,17].

Second messengers and calcium–based oscillatory mechanisms may underlie widespread rhythmic processes involved in the control of vasomotion [18,19], gastric motility [20,21,22,23], intercellular Ca^2+^-waves integrating glucose output in liver [15], and pulsatile reciprocal release in blood of glucose, insulin, and somatostatin vs. glucagon in the pancreas, etc., [23,24,25]. However, many missing links remain in the mechanisms that orchestrate intracellular rhythmicity and intercellular waves’ propagation, despite their extreme importance.

These rhythmic processes are compromised in the course of obesity and type 2 diabetes (T2D) development, indicating impairment of the functions (deregulation of signaling and metabolic pathways) of interstitial cells of Cajal, vascular, and pancreatic α, β, and δ cells, which is manifested as diabetic gastropathy, hypertension and atherosclerosis, and insulin and glucagon resistance, respectively [21,25,26,27,28]. Additionally, hypertension is characterized by acetylcholine resistance and accompanied by the loss of fast and slow rhythmic contractions in aortas of obese and diabetic animals [29]. In addition, limitation of NO-bioavailability [30,31] and NO-resistance [32,33] are often considered important features of obesity, hypertension, and T2D and analyzed separately to rhythmic processes and intracellular Ca^2+^-oscillations’ control.

Apparently, first example of PFL operation, resulting in Ca^2+^-oscillations, was presented by Endo and coworkers on skinned muscle fibers in 1970 [34]. The authors discovered Ca^2+^-induced Ca^2+^-release (CICR) encoded by a ryanodine receptor (RyR), which represents short PFL:

Ca^2+^ → RyR → Ca^2+^ (1)

In 1976, Ridgway and Durham registered Ca^2+^-oscillations in Physarym P. [35]. Ten years later, Woods et al. demonstrated that phenylephrine (PE) and vasopressin (VP) induced Ca^2+^-oscillations in single hepatocyte [7]. Soon after, Thomas and coauthors registered simple and complex Ca^2+^-oscillations, triggering phenomena, and Ca^2+^-waves in hepatocytes induced by PE, VP, NO donor SNAP, and 8-Br-cGMP [8,36]. At this time, Ca^2+^-oscillations were registered in various types of non-excitable and excitable cells [17].

Suggested basic mechanisms of Ca^2+^-oscillations were mainly attributed to [17,36,37,38,39,40]:

- short PFL based on CICR encoded by inositol-3-phosphate (IP_3_)-dependent Ca^2+^-receptor (IP_3_R):
  Ca^2+^ → IP_3_R → Ca^2+^ (2)
- long PFL based on feedback activation of phospholipase C (PLC) by Ca^2+^ and subsequent activation of IP_3_R by the product of PLC reaction IP3:
  Ca^2+^ → PLC → IP_3_ → IP_3_R → Ca^2+^ (3)

At this point, we have to outline that previous theoretical investigations into the analysis of the mechanisms of Ca^2+^-oscillations and Ca^2+^-spikes have been largely focused on the modeling of PLC and IP_3_R- dependent positive feedback mechanisms [15,16]. Last PLC/IP_3_R-centric model includes, in addition, negative feedback based on the inhibition of IP_3_R by Ca^2+^-excess (bell-shaped CICR) [41]. Below, we will indicate the PLC-dependent generator as PLC-G.

However, significant evidence suggests the involvement of lipid kinase γ (PI3Kγ), the NO/protein kinase G (PKG1) signaling system, and cyclic ADP-ribose (cADPr)/RyR in Ca^2+^-spiking and rhythmic processes in various types of cells [5,6,8,9,10,11,12,13,18,22]. Apparently, the matter of overlooking NO-dependent mechanisms is related to the high number of steps between the initial signal input and feedback signal return.

An alternative to the PLC-G oscillatory mechanism, driving the propagation of NO and Ca^2+^-waves in colonic interstitial and smooth muscle cells, was proposed in 1993 [41]. The mechanism was based on the identified mutual amplification of Ca^2+^- and NO-signaling (Ca^2+^→ NO → Ca^2+^) and known activation of constitutive NO synthases (NOS) by Ca^2+^ [42]. The missing link between NO and RyR was found at just the same time, when Gallione and coauthors, working with sea urchin eggs, discovered the modulation of RyR-encoded CICR by cADPr formed, in turn, in NO/soluble guanylate cyclase (sGC)/PKG1)/ADP-Ribosyl Cyclase (ARC)-dependent pathway [43,44]:

NO → sGC → cGMP → PKG1 → ARC → cADPr → RyR → Ca^2+^ (4)

Soon after, operation of this Ca^2+^-signaling pathway was demonstrated on different types of cells. In 2001, Looms et al. reported functioning of this signaling system in rat parotid acinar cells [45,46]. Meanwhile, Zhang et al. registered a RyR-dependent spike-like rise of NO, cADPr, and Ca^2+^ in isolated endothelial cells stimulated by bradykinin (BK) [5]. Taking all these results together, we might reconstruct the main element of NO-dependent generator of rhythmicity (of NOS-G), that is, long PFL implicating Ca^2+^, NO, cGMP, cADPr, and RyR:

Ca^2+^ → NOS → NO → sGC → cGMP → PKG1 → ARC → cADPr → RyR → Ca^2+^ (5)

Following this logic and applying inhibitory analysis, we have shown that acetylcholine (ACh) evoked Ca^2+^ and NO-oscillations and triggering phenomena in cultured white adipocytes of mice involving PI3Kγ-dependent signaling pathway as the input signaling axis for NOS-G. In contrast to the well-known effects of ACh on Gαq→ PLC input axis, ACh, activating muscarinic *m3* cholinoreceptors, switched on the NOS-G requiting input signaling axis [47]:

Gβγ → PI3Kγ → protein kinase B (PKB) → endothelial NOS (eNOS)(6)

In 2003, the activation of this input axis by ACh was disclosed in cardiomycytes [48]. At just the same time, this mechanism was demonstrated in endothelial cells for endothelin-1 (ET-1) [49], VEGF [50], insulin [51], and ACh [52]. Meanwhile, Fisher et al. found that activation of pancreatic acinar cells with bile acids (BA), ACh, and cholecystokinin (CCK) evoked Ca^2+^-responses implicating PI3Kγ and RyR [53]. Collectively, we may suggest that both signaling systems, that is, long PFL (5) and the input signalling axis (6), might operate in various types of cells providing periodic and/or spike-like regimes and triggering phenomena.

In our experiments, norepinephrine (NE), in contrast to ACh, induced rhythmic regimes implicating Gαq and PLC-G [54], while angiotensin II (AngII) switched on NOS-G or PLC-G depending on the cultured adipocytes’ state [55]. RT-PCR analysis revealed the expression of mRNA for all proteins of both signaling systems, supporting the results of inhibitory analysis about the functioning of NOS-G in adipocytes [56].

We consider cultured adipocytes as the simplest model to the study the mechanisms of self- and cross-control of Ca^2+^ signaling systems involved in the generation of rhythmic processes in animal cells.

Here, we investigate the impact of cell size of cultured cells on rhythmicity; the role of influx signaling axes recruited by ACh, NE, AngII, CCK, BK, and insulin (Ins) acting via GPCR and RTK; receptors’ interplay at sub-threshold concentrations of hormones applied; the mechanisms of self-control based on the amplification of main PFL by other kinase-dependent loops; and the impact of obesity on the regulation of PLC-G and NOS-G.

## 2. Results

### 2.1. Diversity of Ca^2+^-Responses Evoked by Various Hormones in Cultured Adipocytes

**Figure 1:**
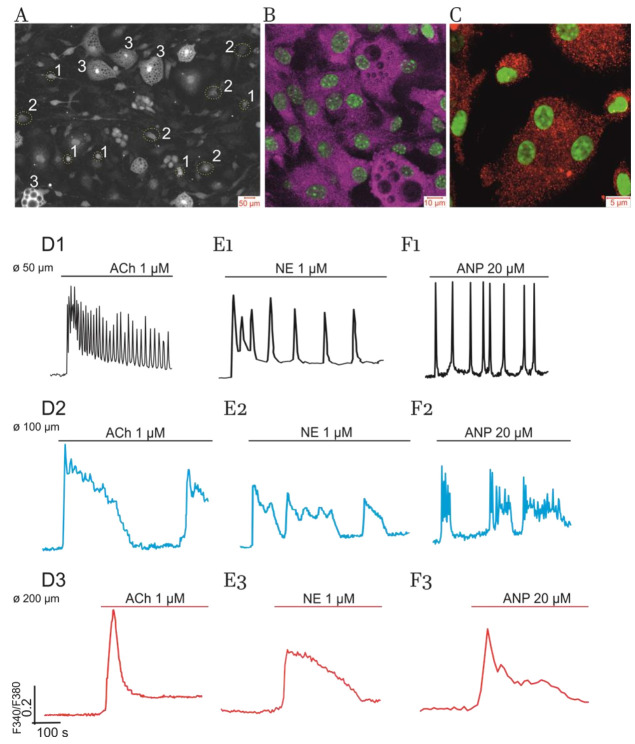
Primary culture of adipocytes derived from eWAT of control mice, expression of ER Ca^2+^-signaling receptors, and Ca^2+^-responses evoked by ACh, NE, and ANP. (**A**) Mature cultured adipocytes (9 DIV) may be separated into three types of cells, based on their size (Ø): 1—cells with small lipid inclusions having diameter Ø ≥ 50 µm and occupying 10–15% of all cells; 2—50% to 60% adipocytes having several lipid droplets and intermediate size of Ø ≥ 100 µm; 3—the cells (15–20%) completely filled with lipids (i.e., hypertrophied “obese” cells) having Ø ≥ 200 µM. Bright field microscopy. See Methods for details. Representative culture with number of the cells in dish (in the field of observation) N = 100–120. (**B**,**C**) Immunocytochemical staining with confocal microscopy characterizing distribution of the proteins of ER Ca^2+^-receptors: IP_3_R (subtypes 1 and 2) were visualized in violet at panel B; RyR (subtypes 2 and 3) were visualized in red at panel C. The nuclei were colored in green. See Methods for details. (**D**–**F**) Impact of cell size. The Ca^2+^-responses evoked by ACh (panels **D**1–**D**3), NE (panels **E**1–**E**3), and ANP (panels **F**1–**F**3) in cultured adipocytes (9 DIV). The traces shown in black (**D**1,**E**1,**F**1) characterize small-size cells (type 1). The responses of intermediate size adipocytes (type 2) are shown in blue. Red traces display the responses of hypertrophied “obese” cells (type 3). Here, representative traces of the changes in cytosolic [Ca^2+^]_i_ (registered as Fura-2 340/380 ratio) are depicted. Number of experiments for each panel: n = 4 to 6. The number of the cells in each culture (in the field of registration): N = 100–120. Percentages (%) of the cells in culture responding in a similar way are indicated at respective traces.

Previously, we have shown that white preadipocytes, isolated from eWAT of healthy mice, actively proliferate in cell culture up to the fifth day and form a monolayer and become differentiated within the next four days (9 DIV) [47]. Figure 1 (panel A) demonstrates that population of cultured adipocytes (9 DIV) is heterogeneous with respect to cell size. These cells are very sensitive to the application of various hormones by generating simple and complex Ca^2+^-oscillations, fast spikes and slow humps, a smooth or steep [Ca^2+^]_i_ rise, and triggering phenomena. Below, we will show that the diversity of responses depends on the type of hormone (receptors) involved, positive feedback Ca^2+^-signaling system engaged, and morphological heterogeneity, which may be characterized by cell size, lipid droplet number, and, apparently, the volume of cytoplasm.

Adipocytes, like most other type of cells, express key proteins of PLC-G and NOS-G, that is, two types of intracellular Ca^2+^ release channels located on the membrane of endoplasmatic reticulum (ER), including subtypes 1 and 2 of IP_3_R and 2 and 3 of RyR (Figure 1, panels B and C). PCR analysis, performed on cultured adipocytes and eWAT tissue samples, also revealed mRNA expression of these receptor isoforms and other proteins involved in the operation of PLC-G and NOS-G [56]. Collectively, these data confirm the results of previous investigations based on inhibitory analysis [47,55].

#### 2.1.1. Impact of Cell Size

Herein, we have found that, depending on cell size, the population of mature adipocytes in culture may be separated into three types of cells with distinct Ca^2+^ responses (Figure 1A):

(1) in total, 10% to 15% of all cells in culture having small lipid inclusions (discernible with specific staining by Oil Red) and size (Ø) equal to or higher than 50 µM (Ø ≥ 50 µM);
(2) in total, 50% to 60% of cells having several lipid droplets and Ø ≥ 100 µM;
(3) the rest of the cells (15–20%) representing hypertrophied “obese” adipocytes completely filled with numerous lipid inclusions and having Ø ≥ 200 µM.

Figure 1 (panels D1, E1, and F1) demonstrates that the cells with small lipid inclusions (type 1) may generate fast [Ca^2+^]_i_-oscillations in response to the application of ACh, NE, and atrial natriuretic peptide (ANP), respectively. Enlarged cells, having intermediate lipid inclusions (type 2), display predominantly slow complex [Ca^2+^]_i_-oscillations (panels D2, E2, and F2). Finally, “obese” adipocytes, having limited volume of cytoplasm (type 3), lose the ability to produce rhythmic patterns and display spike-like [Ca^2+^]_i_ responses to ACh, NE, and ANP.

**Figure 2:**
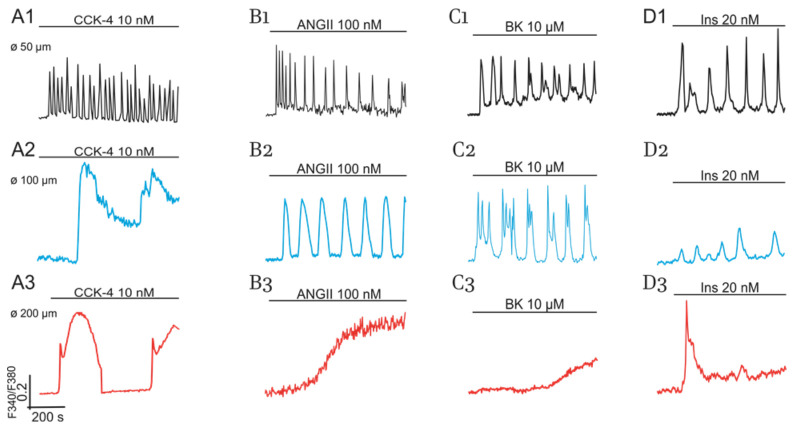
Effects of CCK (**A**1–**A**3), AngII (**B**1–**B**3), BK (**C**1–**C**3), and Ins (**D**1–**D**3) on Ca^2+^- responses of cultured adipocytes (9 DIV) of control mice. Impact of cell size. Responses of small and intermediate size cells and of hypertrophied cells are indicated in black, blue, and red colors, respectively. All conditions of the experiments and abbreviations correspond to Figure 1.

Peptide hormones CCK, AngII, BK, and insulin (Ins) induce similar cell size-dependent effects (Figure 2). Moreover, AngII and BK evoke smooth elevation of [Ca^2+^]_i_ in part of the “obese” cells (Figure 2, panels B3 and C3) compared to the spike-like responses induced by Ach, NE, ANP (Figure 1, panels D3, E3, and F3), and Ins (Figure 2, panel D3).

Notably, at higher concentrations, no hormones can restore rhythmic activity, indicating the development of general hormonal signaling resistance in hypertrophied adipocytes (type 3 cells). Hence, we may suggest that, in addition to the effect on affinity of the receptors to agonists, altered activities (deregulation) of positive feedback Ca^2+^-signaling systems, translating incoming GPCR and RTK signals, may also contribute to the mechanisms of general signaling resistance development.

Results presented at Panels B and C demonstrate a higher level of IP_3_R protein expression than RyR proteins. Based on these results, we might erroneously conclude that the operation of positive feedback Ca^2+^-signaling system targeted to IP_3_R, i.e., PLC-G, may dominate over NOS-G targeted to RyR. However, this is not quite a correct statement. To issue the problem on the implication of PLC-G and NOS-G in various modes of Ca^2+^ responses, mediated by GPCR and TRK, we applied inhibitory analysis.

#### 2.1.2. Impact of GPCR and RTK and Cultured Cells’ State on Switching on of PLC-G and NOS-G

Summary data of our previously published results [47,54,55] and recent inhibitory analysis are presented in Table 1, which describes the impact of the Gαq, Gβγ, and Gα subunits of heterotrimeric G proteins and membrane and cytosolic tyrosine kinases (TK, cSrc) on the control of NOS-G and PLC-G.

In the table: first row indicates hormones applied; second row indicates receptors and subunits of G proteins and tyrosine kinases involved; third and fourth rows indicate Ca^2+^-signaling systems switched on by respective agonist. Numbers (blue and green) characterize the percent (%) of the cells in culture generating mono- or multi-modal oscillations. Minimal and maximal periods of [Ca^2+^]_i_-oscillations, observed in the cells of different size, are presented in the fifth row. In each culture, 5% to 10% of all cells were non-responsive. The rest of the cells produced Ca^2+^-spikes. The number of experiments for each column was 10 to 12. The number of monitored cells in each culture was 80 through 100 cells. The data presented in the table are based on the analysis of previously published results [47,54,55] and recent experiments. Hormones, GPCR, RTK, and proteins are involved in the switching on of PLC-G and NOS-G.

According to data presented in first column, ACh, activating m3-muscarinic receptor (Gq proteins) evoked NOS-G dependent [Ca^2+^]_i_-oscillations in 70–80% of the cells and spike-like effects in the rest of cells. Stimulation of the PI3Kγ and PKB signaling axis (Gβγ→PI3Kγ→PKB→eNOS) underlies this effect. As for the well-known and expected activation of PLC-G by Gαq subunits of Gq proteins (Gαq→PLCβ,γ), the effect was not observed for ACh, apparently, owing to the inhibition of PLC-G via PKG1 phosphorylation of some its proteins (see below).

On the contrary, NE (column 2), acting via α1-adrenoreceptors (Gq proteins), provided PLC-G dependent [Ca^2+^]_i_-oscillations in 30–40% of cells by activating the Gαq→PLCβ,γ signaling axis.

Selective agonists of α2-adrenoreceptors (Gi proteins) induced rhythmicity in an ACh-like manner [47], while the agonists of β-adrenoreceptors (Gs proteins) produced only steep elevation of [Ca^2+^]_i_ [54]. Atrial natriuretic peptide (ANP) induced rhythmic processes in 30–40% of cells by stimulating membrane GC (mGC) and then NOS-G via Gα→mGC→cGMP→PKG1 signaling axis (column 3).

Depending on the used cellular culture, peptide hormones AngII, CCK, and BK (columns 4 to 6) activated PLC-G or NOS-G by implicating Gαq or Gβγ subunits of Gq proteins and PLCβγ or PI3Kγ, respectively. Ins evoked a similar culture-dependent effect. Covalent modification of PI3Kα and PLCβγ by TK and cSrc, respectively, provided a dual effect of insulin (column 7).

**Figure 3:**
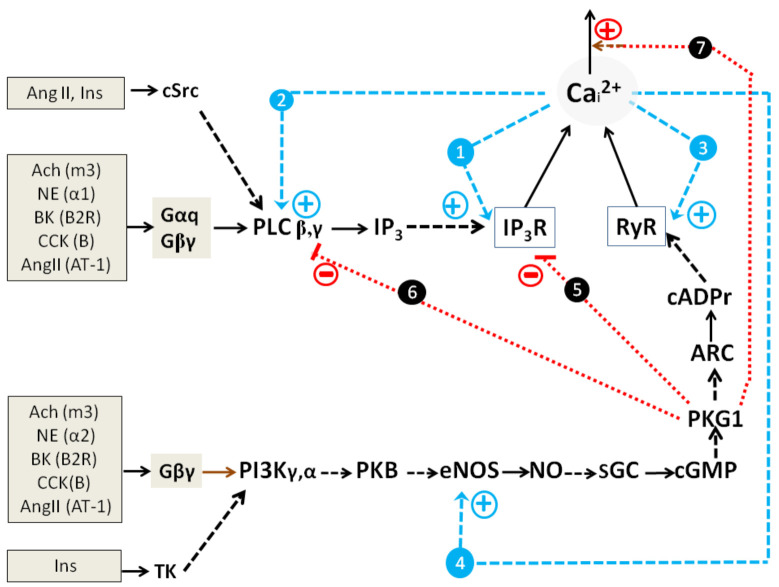
A simplified kinetic model of PLC-G and NOS-G with appropriate input signaling axes, recruiting GPCP and RTK in adipocytes. Various types of activation and inhibition (direct regulation or covalent modification) are indicated as broken arrows and dotted lines (with symbol T), respectively. Positive (blue) and negative (red) feedbacks are numbered 1–4 and 5–7, respectively. Various hormones, respective receptors, G-proteins, and RTK are placed in the boxes. ER Ca^2+^-receptors IP_3_R and RyR are depicted as trapezes. Activation of endoplasmic reticulum and plasmalemmal Ca^2+^-pumps SERCA and PMCA, respectively, via PKG1 is depicted for simplicity as one negative feedback loop 7 (red dotted arrow). Other details are in the text.

Collectively, basic mechanisms of Ca^2+^ oscillations founded on the action of short and long PFLs for PLC-G and NOS-G (Introduction; equations 2,3 and 5,6, respectively) and the above-described GPCR and RTK input signaling axes may be combined in a kinetic model describing the self-control and external regulation of NOS-G and PLC-G (Figure 3).

#### 2.1.3. Simplified Kinetic Model of NOS-G and PLC-G with Input Signaling Axes

Figure 3 depicts both Ca^2+^-signaling systems with their main positive and negative feedbacks and aforementioned input signaling axes. Here, PLC-G and NOS-G have distinct similarity with respect to self-control. Both IP_3_R and RyR possess a fundamental property based on Ca^2+^-induced Ca^2+^-release (CICR). These CICR mechanisms shape two short PFLs (blue broken arrows 1 and 3). Additionally, second messengers IP3 and cADPr reinforce the gaiting of IP_3_R and RYR, respectively. These second messengers facilitate the binding of Ca^2+^ and channels opening (blue broken arrows). In turn, the activation of PLC and eNOS by Ca^2+^ underlies the functioning of two long PFLs (blue broken arrows 2 and 4) that control the values of IP_3_ and cADPr concentrations. PKG1-dependent mechanisms, focused on the activation of [Ca^2+^]_i_ extrusion by Ca^2+^-ATPases of endoplasmic reticulum (SERCA) [57,58,59] and plasmalemma (PMCA) [60,61,62], form negative feedback loops (NFLs) in the system, which are depicted for simplicity as one main NFL (dotted red arrow 7). Thus, both signaling systems represent positive feedback systems (generators) with the families of nested PFLs. In addition, the activities of PLC (isoforms β, γ) and eNOS are controlled by Gαq/cSrc and Gβγ/TK signaling axes, respectively.

The inhibition of PLCβ,γ and IP_3_R, provided by the phosphorylation of RGS (regulator of G protein signaling) and IRAG (IP_3_R-associiated protein kinase G substrate) proteins by PKG1 [63,64], represent negative cross-control (dotted red lines 5 and 6) that ultimately affords switching off PLC-G by ACh. As for the NE effect, a higher gain of the Gαq→PLCβ,γ input axis, compared to the Gβγ→PI3Kγ→PKB→eNOS axis gain, may, apparently, provide preferential switching on of PLC-G by NE.

Here, we might suggest that dual effects of CCK, AngII, BK, and Ins (Table 1) may realize at culture-dependent variations of the expression of any NOS-G proteins, that is, variations that may provide the activation of PLC-G or NOS-G at low or high activities of PKG1β, respectively. Likely, culture-dependent variations of input axes gains may also underlie dual effects of peptide hormones. L-arginine, the substrate of eNOS, was included in incubation media in all our experiments to avoid the limitation of NOS-G activity by NO availability. Over the last forty years, much attention has been paid to the study of the mechanisms of PLC-G control [15,17]. Below, we will focus on NOG-G regulation.

### 2.2. Combined Synergistic Action of Hormones Implicated in Parametric Control of NOS-RG

**Figure 4:**
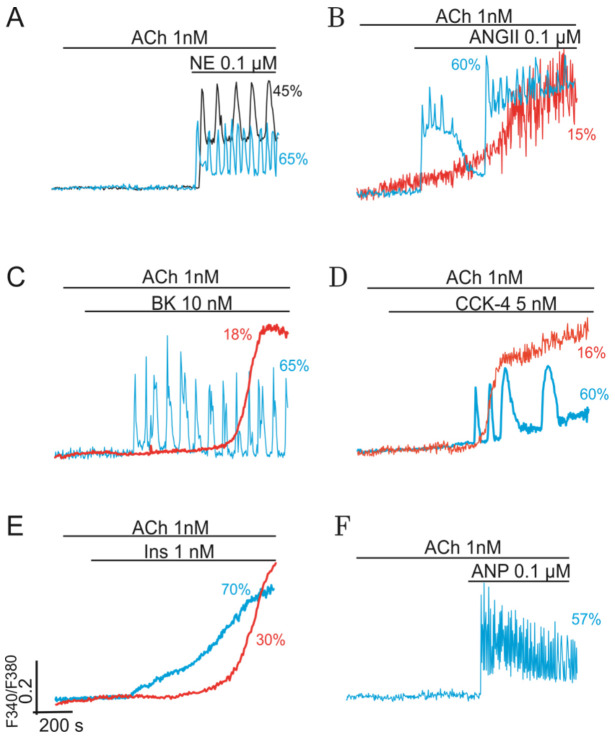
Synergistic effect of ACh (Gq) and NE (Gq) or ACh (Gq) and other hormones on NOS-G. Paired application of hormones, used at sub-threshold concentrations, provides G proteins interplay, signal amplification, and the induction of Ca^2+^-oscillations in most of the cells (representative traces are shown in blue). (**A**) At low sub-threshold concentration 0.1 µM, NE reinforces the effect of 1 nM ACh, providing regular Ca^2+^-oscillations in most cells (blue trace). Black trace corresponds to the experiment carried on after preincubation of the cells with 3 µM U73122, the inhibitor of PLC. (**B**–**E**) Paired effects of ACh and peptide hormones AngII, BK, CCK, and Ins. Except Ins, which evoked a steep rise of [Ca^2+^]_i_ in adipocytes (panel E, blue line), all other hormones reinforced the effect of ACh and induced Ca2+-oscillations in most of cells (blue traces). Red trace at panel E depicts the experiment carried on after preincubation of the cells with 3 µM U73122, the inhibitor of PLC. (**F**) At low sub-threshold concentration 0.1 µM, ANP reinforces the effect of 1 nM ACh, providing regular Ca^2+^-oscillations in most cells (blue trace). All conditions of the experiments and abbreviations correspond to Figure 1. Other details are in the text.

At low sub-threshold concentrations (≤10 nM), ACh, BK, CCK, and Ins (≤2 nM) cannot evoke visible Ca^2+^-responses in cultured cells. Similarly, Ca^2+^-signaling systems of adipocytes do not respond when the concentrations of NE, ANP, and AngII are equal to or lower than 100 nM. However, combined applications of ACh and any of indicated hormones provide the generation of various modes of Ca^2+^-oscillations in most cells (Figure 4, blue traces, ≥50–70% cells). In this case, the interplay of G-proteins of respective GPCRs ensures the needed signaling amplification and switching on of NOS-G. All these effects are preserved in the presence of PLC inhibitors, suggesting the suppression of PLC-G by the impact of PKG1, as was discussed above.

#### 2.2.1. Gβγ-Subunits Interplay

Panel A in Figure 4 characterizes the synergistic action of 1 nM ACh and 100 nM NE, which may be based on the interplay of Gβγ -subunits of G proteins. Here, the combined action of both agonists, added at sub-threshold concentrations, provided the required level of Gβγ→PI3Kγ→PKB→eNOS signaling axis stimulation that, in turn, ensured the activation of NOS-G. The effect is preserved in the presence of U73122 (black vs. bluetraces), a known inhibitor of PLC, which indicates the switching on of NOS-G at the combined action of ACh and NE.

Previously, we have shown that a similar synergistic effect may be observed also for ACh and phenylephrine or selective agonists of α1- or α2-adrenoreceptors [47]. In the last case, the required level of eNOS activation, apparently, was attained by integral action of Gβγ subunits of Gq and Gi proteins on PI3Kγ. Indeed, the impact of NE on NOS-G is not limited by the signaling effects of α-adrenoreceptors. Activation of β-adrenoreceptors may also be involved in switching on of this positive feedback system (see below).

It should be noted here that external activation of eNOS, like any other protein incorporated in long PFL of NOS-G, represents parametric control of this system. In addition to NE, various peptide hormones, being used at low concentrations, may amplify the signaling action of ACh at the level of Gβγ-subunits of Gq proteins and recruit NOS-G. Figure 4 demonstrates that combined action of ACh and AngII (panel B), ACh and BK (panel C), and ACh and CCK (panel D) evoke complex [Ca^2+^]_i_ responses in cultured cells. Fine parametric control of NOS-G, directed to eNOS via the Gβγ→PI3Kγ→PKB→eNOS signaling axis, provides sustained [Ca^2+^]_i_ oscillations in most cells (blue traces) or switching of this system to a new states with high values of [Ca^2+^]_i_ in the rest 10–20% cells (red traces).

#### 2.2.2. Gβγ/TK Interplay

On the contrary, the combined action of 1 nM ACh and 2 nM Ins provided only a steep rise of [Ca^2+^]_i_ in most cells (Figure 4, panel E, red trace). This effect was also preserved in the presence of PLC inhibitor U73122 (red trace), indicating Gβγ/TK interplay and combined action of Gβγ→PI3Kγ→PKB→eNOS and TK→PI3Kα→PKB→eNOS signaling axes on eNOS. Apparently, over-activation of eNOS switched NOS-G in the state with high [Ca^2+^]_i_, preventing rhythmic responses of the system. Certainly, the combined effect of hormones, added at high concentrations, may also lead to similar triggering phenomena characterized by the transition of this positive feedback Ca^2+^-signaling system into the states with high Ca^2+^-concentrations due to excessive activation of long PFL at the level of eNOS.

#### 2.2.3. Gβγ/Gα Proteins Interplay

Like eNOS, cytosolic PKG1 represent the second natural target of parametric control in the system. ANP, activating membrane GC (mGC), may turn on the signaling axis focused at PKG1: Gα→mGC→cGMP→PKG1. At low concentration 100 nM, ANP reinforces the effect of 1 nM ACh, i.e., of the axis directed on eNOS. Combined activation of both axes (i.e., of eNOS and PKG1) provides fine-tuning of NOS-G, evoking rhythmic responses (Figure 4, panel F).

#### 2.2.4. Gβγ/Gs Proteins Interplay

**Figure 5:**
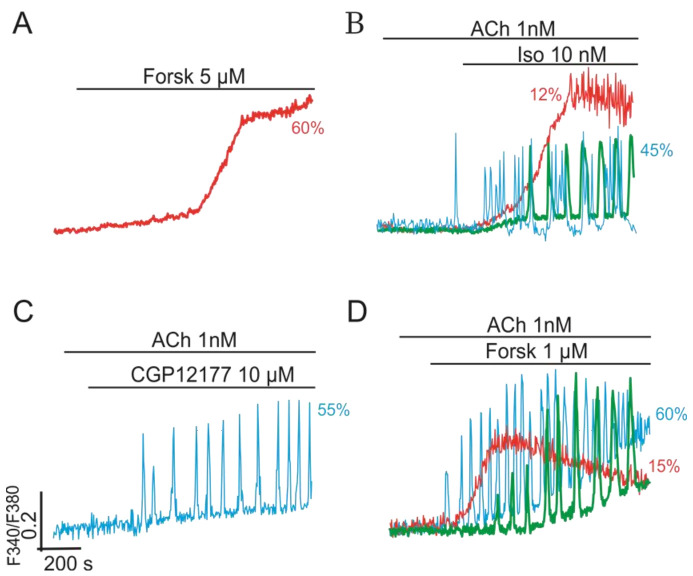
Synergistic effect of ACh (Gq) and the agonists of β-adrenoreceptors (Gs) on NOS-G providing induction of Ca^2+^-oscillations in cultured adipocytes. (**A**) At high concentration 5 µM, activator of AC forscolin, stimulating the Gs→AC→cAMP→PKA→RyR signaling axis, may evoke only a delayed steep rise of [Ca^2+^]_i_. (**B**) At low sub-threshold concentration 1 nM, β-adrenoreceptors agonist isoproterenol reinforced the effect of 1 nM ACh, providing various types of regular and complex Ca^2+^-oscillations in most cells. (**C**) Similarly, the selective agonist of β3-adrenoreceptors CGP12177 (10 µM) amplified the effect of ACh and induced regular modes of Ca^2+^-oscillations in most cells. (**D**)–At low sub-threshold concentration 1 µM, forskolin reinforced the effect of 1 nM ACh on NOS-G, which resulted in the induction of various modes of Ca^2+^-oscillations and triggering phenomena. All conditions of the experiments and abbreviations correspond to Figure 1. Other details are in the text.

Regulation of RyR by various kinases represents the third point of parametric control of NOS-G. Stimulation of β-adrenoreceptors switch on the adenylate cyclase (AC)/protein kinase A (PKA) signaling axis: Gs→AC→cAMP→PKA→RyR. This axis provides control of NOS-G mediated by the phosphorylation of RyR by PKA. Previous studies indicated that activation of this axis by selective agonist of β-adrenoreceptors isoproterenol, permeable analogs of cAMP, or activator of AC forscolin evoked only a delayed steep increase of [Ca^2+^]_i_ in most adipocytes [54], like the effect of 5 µM forskolin shown in panel A of Figure 5. However, being used in low concentration 10 nM, isoproterenol, in combination with 1 nM ACh, provides dual parametric control of NOS-RG, that is, based on the impact of Gβγ→PI3Kγ→PKB→eNOS and Gs→AC→cAMP→PKA→RyR signaling axes on eNOS and RyR, respectively.

Here, dual control of NOS-G results in the induction of complex [Ca^2+^]_i_ oscillations in most cells (Figure 5, panel B, blue trace). Selective β2-adrenoreceptors agonist CGP12177 and 1 µM forscolin evoke similar effects (panels C and D).

### 2.3. Multi-Loop Positive Feedback Control and Robustness of NOS-G

**Figure 6:**
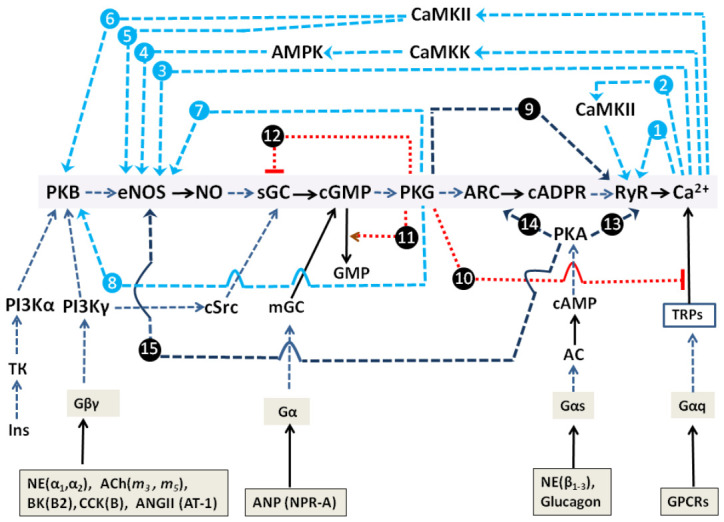
Detailed kinetic model of NOS-G with its multi-loop positive feedback control and input signaling axes. Here, various types of activation and inhibition are indicated as arrows and dotted lines with symbol T, respectively. The family of nested positive feedback loops is depicted as blue arrows numbered 1 to 8 (in blue circles). The positive feed forward loop, based on the phosphorylation of RyR with PKG1, has the number 9. Negative feedbacks (marked by black circles and shown in red) are numbered 10 through 12. Crosstalk loops, describing the positive impact of AC/cAMP/PKA-signaling axis on NOS-RG (on PKB, ARC, and RyR), have the numbers 13 through 15. Various hormones (with corresponding receptors), activating G-proteins and RTKs, are placed in the boxes. Known effects of PKG1 on Ca^2+^-pumps SERCA and PMCA and activation/inhibition of phosphodiesterases II/III by PKG1 are omitted for simplicity. Description of this model and other details are given in the text.

The simplified kinetic model of NOS-G, presented in Figure 3 includes only two PFLs, that is, short and long loops, which are based on the control of RyR and eNOS by Ca^2+^. Although it is well known that several protein kinases are also implicated in the regulation of the proteins of NOS-G. However, the impact of these kinases on the control of NOS-G is not studied in detail yet. Correct inclusion into the kinetic model of the regulatory loops, formed with the participation of these kinases, allows revealing their role in the self-control of the system. The detailed kinetic model of NOS-G, which incorporates the above-mentioned regulatory loops and all input signaling axes, is depicted in Figure 6.

It is well known that covalent modification of eNOS with PKA, PKG1, Ca^2+^/Calmodulin-dependent kinase II (CaMKII), and AMP-dependent kinase (AMPK) may result in the activation of this enzyme [65,66]. AMPK, in turn, is activated via Ca^2+^/Calmodulin-dependent kinase kinase (CaMKK) phosphorylation [67]. Additionally, RyR and PKB are known to be activated via PKG1 and CaMKII-dependent pathways [32,68]. Collectively, all aforementioned kinases are implicated in several PFLs involved in the self-control of NOG-G. All these PFLs are depicted in Figure 6 as broken blue arrows numbered 1 to 8. Red dotted lines 10 and 12 with sign T (indicating inhibition) and red arrow 11 (indicating activation) correspond to negative feedback loops (see for details [56]).

Although much information exists regarding the impact of CaMKII and AMPK on target proteins, very little is known about the contribution of respective PFLs 4 to 6 (Figure 6, blue arrows 4–6) in the control of this Ca^2+^-signaling system. To issue this problem, we applied the inhibitors of CaMKII and AMPK, KN62 and Compound C, accordingly. Suppression of the activity of any of these enzymes may, in theory, totally or partially diminish the gain of respective PFLs, depending on the concentration of inhibitor used. Here, we consider the number of the cells in culture (N), responding rhythmically to ACh as a measure of efficient positive feedback control of NOS-RG.

**Figure 7:**
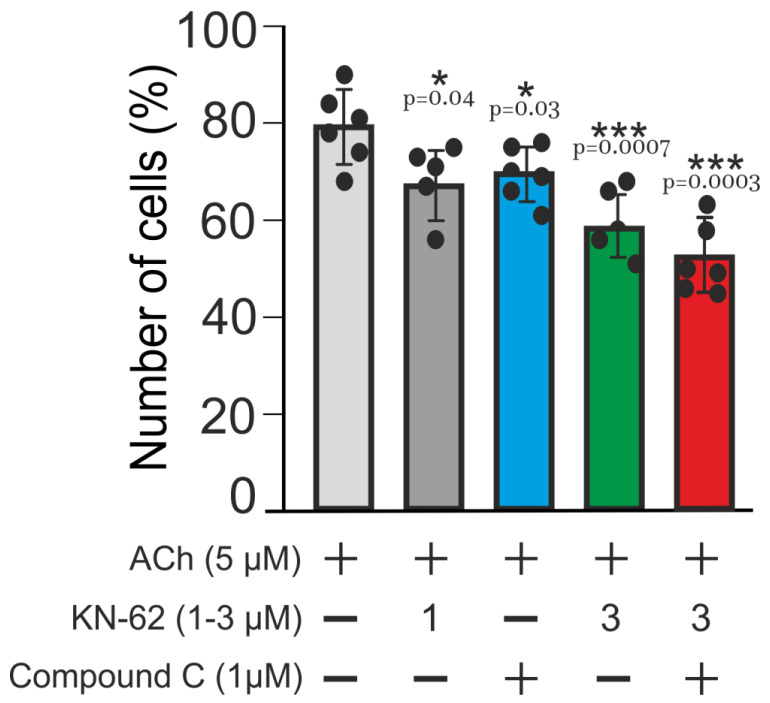
Impact of CaMKII and AMPK on Ca^2+^-oscillations elicited by ACh in adipocytes and robustness of NOS-G. Bars represent average number of the cells, which respond to added ACh in control (black bar) and in the presence of the inhibitors of CaMKII (KN-63) and AMPK (Compound C). Concentrations of ACh and inhibitors are depicted in the figure. Sign + indicates the application of ACh or of the inhibitors. The inhibitors were added 10 min before the application of ACh. All conditions of the experiments and abbreviations correspond to Figure 1. Details are given in the text. For each column, the number of experiments n = 6. Data presented as mean ± SE.

Figure 7 shows that ACh evoked Ca^2+^-oscillations in up to 79% cells in the control (black column). The inhibitors were added 10 min before the application of ACh. At low concentration 1 µM, nearly equal to IC50 for CaMKII, KN62 provided partial suppression of respective PFLs diminishing N to 68% (gray column), which was statistically insignificant. Similarly, at 1 µM, Compound C (IC50 ~ 0.1 µM for AMPK) produced a weak effect by decreasing the rhythmicity to 70% (blue column). Combined application of both inhibitors suppressed the rhythmicity by 25%, decreasing N to 59% (green column). Applied at 3 µM, KN62, apparently, substantially inhibited CaMKII activity and diminished the gains of respective PFLs (Figure 6, arrows 2, 4–6). The number of oscillating cells in culture N decreases to 59% (Figure 7, green column) indicating a marked contribution of CaMKII in the self-control of NOS-G. We did not use KN-62 at a higher concentration to avoid unspecific effects. A weak effect of Compound C on NOS-G might be explained by the fact that the activation of AMPK by CaMKK was insufficient to keep the required amplification of PFL4 (Figure 6, arrow 4) at the conditions of our experiments. It is known that, besides CaMKK, AMP, sirtuins, and several kinases control the activity of AMPK [67]. A marked effect of the combined application of 3 µM CaMKII and 1 µM AMPK inhibitors (red column) may be based on cross-talk between these enzymes by yet unknown mechanism.

Results presented in Figure 7 demonstrate that manipulations with the gain of some PFLs did not alter systemic response dramatically; most of the cells preserved rhythmicity evoked by ACh (56–59% vs. 79% oscillating cells in control). Here, we may suggest that strengthening of core PFL (arrow 3) by other PFLs (arrows 2 and 4–8) underlie the reliability of NOS-G operation.

### 2.4. Impact of Obesity on Cultured Adipocytes

To evaluate the effect of obesity on cell growth, lipid accumulation, and rhythmic activity, we used long-term high-fat feeding, taking in the experiments 8-week-old mice. A brief description of this model and the values of blood glucose, insulin, lipids profile, and blood pressure are presented in Methods (Table 2). Obese 7-month-old overweight mice (42.5 ± 0.72 vs. 24.4 ± 0.5 g; SE) had elevated levels of glucose (8.01 ± 0.21 vs. 6.4 ± 0.15 mM) and insulin (1.95 ± 0.11 vs. 0.51 ± 0.04 ng/mL) in blood in a fasted state, and raised arterial pressure (143 ± 1.5 vs. 121 ± 2.4 mm Hg).

#### 2.4.1. Effect of Obesity on Adipocytes Lipids Accumulation and Expression of IP_3_R and RyR

**Figure 8:**
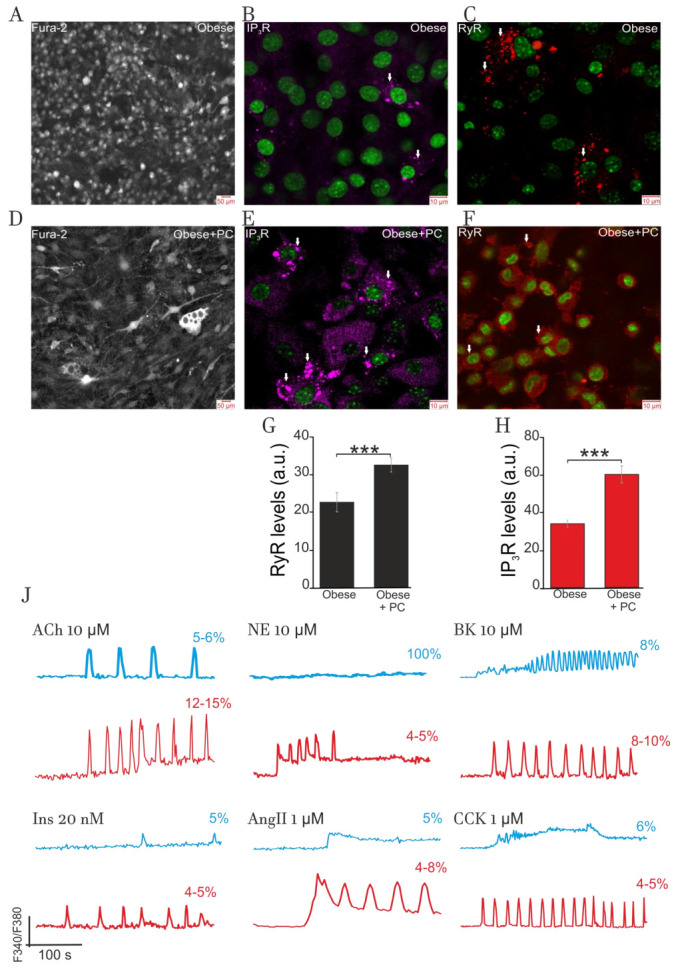
Impact of obesity on lipid accumulation, IP_3_R and RyR expression, and Ca^2+^-signaling in cultured adipocytes. (**A**) Bright field microscopy. Cells, obtained from preadipocytes isolated from eWAT of obese mice (7 months old) and growing on high glucose DMEM media, have small size and cannot enlarge and accumulate lipids. (**B**,**C**): These cultured adipocytes have low level of the expression of IP_3_R and RyR proteins and show some clustering of both receptor proteins (arrows). (**D**) Bright field microscopy. Inclusion 100 nM palmitoyl-L-carnitine in the media, since the second day of incubation, provided the accumulation of lipids in 10% to 15% of 9DIV cells (arrows). (**E**,**F**): Added palmitoyl-L-carnitine (100 nM) afforded the raise of the expression of IP_3_R and RyR proteins compared to cells cultured in glucose DMEM. Additionally, 100 nM palmitoyl-L-carnitine increased clustering of both receptor proteins (arrows at Panels (**E**,**F**)). (**G**,**H**): Bars, shown at panels (**G**,**H**), indicate about 2-fold increases in the expression of these receptor proteins registered in the presence of palmitoyl-L-carnitine. At the panels (**B**,**C**,**E**,**F**), immunocytochemical staining with confocal microscopy was applied. (**J**) Effect of hormones on cytosolic [Ca^2+^]_i_-signaling in adipocytes grown on high-glucose DMEM media (panel J, blue traces) and on high-glucose DMEM+ 100 nM palmitoyl-L-carnitine media (panel **J**, red traces). Applied at high concentrations, NE, CCK, BK, and Ins did not evoke Ca^2+^-oscillations in the first type of cells (blue traces). In contrast, ACh and BK induced rhythmic activity in 5–6% of cells and spike-like response in 8–10% of cells (not shown). Added in media at 100 nM, palmitoyl-L-carnitine partially restored Ca^2+^-rhythmicity for ACh (12–15% of all cells) and BK (8–10%), while respective values for NE, CCK, AngII, and Ins did not exceed 5% (panel **J**, red lines). All conditions of the experiments and abbreviations correspond to Figure 1. Other details are in the text. *** *p* < 0.001.

Figure 8 characterizes cultured adipocytes prepared from the preadipocytes of eWAT of obese mice. Cells, growing on high glucose DMEM media, have small size and cannot enlarge and accumulate lipids, apparently, due to the low level of acetyl-CoA carbohylase expression and impaired synthesis of fatty acids from glucose in this model of obesity (panel A, 9DIV). These cells also have low expression of the proteins of ER Ca^2+^-receptors IP_3_R (Panel B) and RyR (panel C) and display moderate clustering of both types of receptors (panels B and C, arrows). Preliminary data indicate that clustered receptors are nonfunctional.

However, the inclusion of 100 nM palmitoyl-L-carnitine in the incubation media provided the accumulation of lipids in 10% to 15% of cells (panel **D**, 9DIV, arrows). Surprisingly, cells grown in the presence of palmitoyl-L-carnitine had raised expression of IP_3_R and RyR proteins (panels E and F) compared to the cells cultured in glucose DMEM. Bars, shown at panels G and H, indicate about a two-fold difference in the expression of these receptor proteins. Additionally, even at low 100 nM, palmitoyl-L-carnitine increased clustering of both receptor proteins (arrows at panels E and F).

#### 2.4.2. Impact of Obesity on Ca^2+^-Signaling

Panel J depicts the effects of hormones on Ca^2+^-signaling in the cells (9DIV) cultured on glucose (black traces) and glucose + palmitoyl-L-carnitine media (blue traces). Cells grown on glucose media demonstrated very weak Ca^2+^-signaling (red traces). Added at a high concentration of 10 µM, ACh evoked [Ca^2+^]_i_ oscillations in 5–6% cells and spike-like responses in 8–10% cells (not shown). A similar effect was produced by 10 µM BK. Induction of rhythmic processes in these cells may indicate the functioning of NOS-G and PLC-G responsive to ACh and BK, respectively. In contrast, being used at high concentrations of 1 µM, AngII and CCK induced only smooth elevation of [Ca^2+^]_i_ in this limited number of cells (4–5%). At this point, we may suggest that impaired (suppressed) activities of respective input signaling axes might underlie insufficient stimulation of NOS-G or PLC-G by AngII and CCK to induce [Ca^2+^]_i_ oscillations. These cells do not respond to NE (10 µM) and Ins (0.02 µM). This fact may indicate drastic deregulation of respective input signaling axes, including impaired GPCR and TK-receptor expression and/or drastic alteration in the receptor affinities to respective agonists.

Growth of adipocytes in the presence of low 100 nM palmitoyl-L-carnitine provided the restoration of signaling sensitivity to all hormones in 5% to 15% of the cells, depending on the hormone used. This effect is predominantly registered in the cells accumulating lipids. At high concentrations, ACh, NE, and peptide hormones induced [Ca^2+^]_i_ oscillations in these cells (panel J, red traces) that may indicate the functioning of NOS-G and/or PLC-G. However, compared to adipocytes derived from eWAT of healthy animals (Table 1), the number of the cells sensitive to applied hormones was several times lower at this stage of obesity.

Here, we might speculate that IP_3_R and RyR clustering and ER stress may also contribute to deregulation of both Ca^2+^-signaling systems and lowering the number of adipocytes producing rhythmic and spike-like Ca^2+^-responses. As for the effect of palmitoyl-L-carnitine, long chain fatty acids or their derivatives, like ceramides, may have an impact on various genomic targets to alter the expression and profiles of activities for the proteins of various metabolic and signaling systems.

In our model of obesity (Table 2 in Methods), 8-to-9-month high-fat animal feeding results in the development of T2D, which is characterized by high glucose (11.9 ± 0.4 mM) and Ins (3.4 ± 0.1 ng/mL) in the blood and high AP (177 ± 2.9 mmHg). At this final stage of obesity, most cultured cells do not respond to any hormones (not shown). This non-responsiveness may indicate severe impairment of the expression for the proteins of various metabolic and signaling systems in preadipocytes derived from eWAT. Similar hormonal resistance was registered in primary hypertrophied adipocytes derived from eWAT of diabetic mice. These cells, having 3–5% cytoplasm volume, responded only to calcium or ionophore ionomycin, being not sensitive to the application of 10–20 µM ACh and NE or peptide hormones [56].

Collectively, we may suggest that cultured and primary adipocytes, derived from obese mice, demonstrate general hormonal resistance, which has some similarities with the hormonal resistance observed in hypertrophied “obese” cells of healthy animals (Figure 1).

## 3. Discussion

Here, we used cultured adipocytes as a simple convenient model to investigate the mechanisms of self- and cross-control of Ca^2+^-signaling systems involved in the generation of short-term rhythmic processes in animal cells. Simple and complex oscillation, Ca^2+^-spikes, triggering phenomena, and multistability indicate the operation of PFLs in cellular Ca^2+^-signaling systems [16,17]. Multistability may be characterized by the set of steady states with low, intermediate, or high values of Ca^2+^, NO, cGMP, cADPr, and different enzyme activities (velocities) and Ca^2+^ channel states. The above-presented examples of triggering phenomena illustrate this important property of positive feedback systems. Birhythmicity and multiperiodic and chaotic regimes may appear in cellular metabolic and/or signaling systems with two or more PFLs [16]. The values of the system’s parameters (enzyme activities, channel and influx axe’s states, etc.) influence the mode of positive feedback system, determining whether it be a rhythmic, triggering, spike-like, or smooth response. Adipocytes displayed ubiquitous rhythmicity in response to any hormone applied, being most sensitive to ACh, which evoked [Ca^2+^]_i_ oscillation in most of cells (70% to 80%, Figure 1, Table 1).

We evaluated the efficiency of positive feedback control in both Ca^2+^-signaling systems measuring the number of the cells in culture (N) responding rhythmically to any hormone applied. We recognize the convention of such an assessment, since spike-like effects and triggering phenomena belong to positive feedback systems responses. For ACh, 10–15% cells generated [Ca^2+^]_i_ spikes and 5–10% displayed triggering phenomena. Other hormones, presented in Table 1, evoked [Ca^2+^]_i_ spike-like responses in 40–60% cells. However, here, we are focused on the oscillations, because the probability of [Ca^2+^]_i_ oscillation is the measure of Ca^2+^-waves propagation extent in the cells. To avoid the photo damaging effect, arising at high-speed resolution, in present experiments, we did not monitor Ca^2+^ waves requiring a long time of registration.

### 3.1. Positive Feedback Loops Interplay

Figure 1 and Figure 2 demonstrate that ACh, NE, and four peptide hormones evoke complex [Ca^2+^]_i_ oscillations in part of cultured cells. This indicates that the interplay of short and long PFLs of PLC-G or NOS-G may underlie multiperiodic regimes generated by PLC-G or NOS-G in these cells. These PFLs are depicted as PFLs 1, 2 for PLC-G and PFLs 3, 4 for NOS-G in Figure 3.

The interplay of long PFLs in NOS-G fulfils another function. The extended kinetic model of NOS-G, presented in Figure 6, incorporates all input axes and several positive and negative feedback loops involved in the autoregulation of this signaling system. Here, long PFLs 4 to 6 embrace the main PFL3. Based on this fact, we may suggest that PFLs 4 to 6 may raise the total gain of positive feedback in the system, duplicating the operation of long PFL3 (arrow 3). According to this hypothesis, we applied the inhibitors of CaMKII and AMPK (KN-63 and Compound C, respectively) to diminish the strength of PFLs 2, 4–6. As a result of the combined application of both inhibitors, the number of adipocytes, responding rhythmically to ACh, decreased by about 30% (Figure 7, right columns; 57% vs. 79% oscillating cells in control), which demonstrates substantial contribution of PFLs 4 to 6 into the regulation of NOS-G in these 30% cells. However, most of the cells preserved rhythmicity evoked by ACh, indicating robustness of NOS-G. Apparently, total gain in the system may be reinforced by feedback loops 7 and 8 too. However, we cannot evaluate the contribution of these loops in the control of the system by inhibiting PKG1. Obviously, the suppression of PKG1 or any other protein incorporated inside of core PFL3 would destroy its operation. Short PFL2 may reinforce the operation of short PFL1 (of CICR). Collectively, we may suggest that the nested structure of PFLs in NOS-G (Figure 6) provides duplication and reinforcement of the main PFL3 and underlies the reliability of NOS-G operation.

### 3.2. Extreme Sensitivity of NOS-G to Input Signal and G Proteins Interplay

Besides reliability, the nested structure of the positive feedback system (Figure 6) provides its extreme sensitivity to any input signals directed to the elements embraced by PFL3. Figure 4 and Figure 5 demonstrate that the combined application of ACh and NE, used at sub-threshold concentrations, or ACh and other hormones or receptor agonists evoked the switching on of NOS-G, owing to G proteins’ interplay. G proteins’ interplay provides the required activation of input signaling axes focused on the elements of PFL3 (Figure 6). Collectively, we may suggest that NOS-G is a very sensitive multivariable positive feedback signaling system integrating input signals and generating diverse [Ca^2+^]_i_ responses depending on the combinations of hormones used.

Similar input signal amplification and G protein interplay may realize for PLC-G. In 2019, Thomas et al. demonstrated that combined action glucagon and VP, added at sub-threshold concentrations, provided synchronization of intercellular Ca^2+^ waves and raised glucose output by the perfused liver [22].

### 3.3. Over-Activation of Input Axes and Multistability of NOS-G

Combined activation of eNOS by ACh and Ins via Gβγ→PI3Kγ→PKB→eNOS and TK→PI3Kα→PKB→eNOS signaling axes, respectively, prevented [Ca^2+^]_i_ oscillations and transferred the system into the state with high [Ca^2+^]_i_ (Figure 4, panel E), apparently, due to over-activation of eNOS. This effect was observed for all cells, demonstrating that a delicate balance of enzyme activities is required for rhythmic operation of NOS-G. Like triggering effects were registered for Ach and BK (or CCK and AngII) only in 10% to 18% of cells (panels B–D).

Figure 5 also indicates that gentle activation of the PKA-dependent input axis (Gαs→AC→cAMP→PKA→RyR) evoked triggering of NOS-G into the state with high [Ca^2+^]_i_ only in 10% to 20% cells, preserving [Ca^2+^]_i_ oscillations in the rest of cells. The probability of such transitions rose with the concentrations of agonists applied (not shown). The same is true for the combined action of ACh (1 nM) and ANP (100 nM). At sub-threshold concentrations, both hormones switched on NOS-G, evoking [Ca^2+^]_i_ oscillations (Figure 4, panel F). However, over-activation of the Gα→mGC→cGMP→PKG1 signaling axis by ANP may induce triggering phenomena founded on positive and negative feedbacks’ crosstalk.

### 3.4. Positive and Negative Feedbacks’ Crosstalk

In 2013, we have shown that 1 µM ANP induced complex oscillations, while sequential application of 1 nM ACh and 1 µM ANP caused transient oscillations with the following transfer of the system into the state with high [Ca^2+^]_i_. In contrast, combined action of ACh (5 nM) and ANP (10 µM) evoked damped [Ca^2+^]_i_ oscillations with the transfer of NOS-G into the state with low [Ca^2+^]_i_ [47] indicating reinforcement of the main negative feedback loop (arrow 7 at Figure 3) based on the impact of PKG1 on Ca^2+^ extrusion by Ca^2+^-ATPases (SERCA and PMCA).

Two examples presented below may further illustrate the crosstalk of PFL3 and negative feedbacks. In 1998, Thomas and coauthors have demonstrated dose-dependent effects and triggering phenomena in isolated hepatocytes at the combined application of PE and 8-br-cGMP, the cell-permeable analog of cGMP. At a low concentration of 200 µM, 8-br-cGMP induced [Ca^2+^]_i_ oscillations in silent cell pretreated with PE. Meanwhile, at 1 mM, 8-Br-cGMP suppressed [Ca^2+^]_i_ oscillations evoked by PE and switched the Ca^2+^ signaling system into the state with high [Ca^2+^]_i_ [8], which may indicate the dominant role of PFL3 in the control of Ca^2+^ homeostasis in this state.

At just the same time, bimodal effects of C-natriuretic peptide (CNP) on cardiac contractility were observed on ventricular strips and isolated hearts of rodents [69,70], suggesting temporal alterations of the NOS-G state, determined by the counterbalance of PFL3 and negative feedbacks. Initial time-dependent activation of PKG1 (via CNP axis) evoked an immediate contractile response mediated, apparently, by the activation of RyR and rise of [Ca^2+^]_I_ and indicating the prevalence of PFLs over NFLs. The slow development of the negative inotropic effect might have to indicate delayed reinforcement of negative feedbacks based on the activation of Ca^2+^ removal by SERCA, PMCA via PKG1 phosphorylation, and by impact of PKG1 on contractile proteins.

### 3.5. Obesity-Induced General Hormonal Signaling Resistance of Adipocytes

Five-month high-fat feeding dramatically altered the responses of cultured adipocytes to all hormones tested. Being adapted to take up fatty acids from the blood, preadipocytes from eWAT of obese mice can synthesize and accumulate lipids only at incubation media containing fatty acids. Cells growing with palmitoyl-l-carnitine in the media partially restored rhythmicity. However, only 5% to 15% of all cells in cultures displayed Ca^2+^-oscillations and spike-like responses, indicating the development of general hormonal signaling resistance and loss of fine positive feedback control in cultured cells of eWAT from obese mice (Figure 8, panel J, red tracks). These alterations have some qualitative similarity to the alteration of Ca^2+^ signaling registered in hypertrophied “obese” cells of control mice, having limited volume of cytoplasm (Figure 1 and Figure 2).

Analysis of mRNA expression of marker genes for the proteins, involved in the operation of NOS-G, PLC-G, and respective input signaling axes, revealed 2–3 times more down regulation of PI3Kγ, CaMKIIβ, AMPK, IP_3_R1, and IP_3_R2 genes and 7–10 times lower expression of PKG1, PKG2, ARC (CD38), and eNOS genes compared to the cells of age-matched control mice. In animals with T2D, developed after eight months of high-fat feeding, no hormones can restore rhythmic activity in cultured or primary adipocytes. The progress of obesity further aggravates marker genes’ expression, especially of NOS-G. The expression of eNOS falls by about 15 times, while the expression of the genes for RyR2 and RyR3 was under the level of detection compared to the age-matched control [56]. Registered disproportionate alterations of marker genes’ expression may indicate disproportionate alterations for the activities of respective enzymes, underlying the final deregulation of both Ca^2+^-signaling systems and respective input axes, displayed as non-responsiveness to hormonal signals.

Here, we may suggest that, besides the diminished affinity of the receptors to agonists, deregulation (disproportionally altered enzyme activities) of Ca^2+^-signaling systems may also contribute to the mechanisms of general signaling resistance development, manifested as impaired translation of incoming GPCR and RTK signals.

## 4. Materials and Methods

All animal procedures were fulfilled in accordance with the EU directive 86/609/EEC. The trial was approved by the Ethics Committee at the Institute of Theoretical and Experimental Biophysics, RAS, Pushino, Russia (Protocol №1, 17 February 2020). Male albino mice were kept under the same conditions in air-conditioned and ventilated rooms at 20–22 °C with a 12 h/12 h light–dark cycle. All experiments were performed at 28 °C.

### 4.1. Isolation and Cultivation of Preadipocytes

Cell cultures were prepared as described in detail previously [47]. Briefly, we used NMRI mice (aged 4–6 weeks). White adipose tissue was removed from the epididymal fat depot and digested with 7 mg collagenase II (Sigma-Aldrich, St. Louis, MO, USA) for 18 min at 37 °C. To stop the enzymatic reaction, the tube was chilled on ice for 20 min with intermittent shaking followed by filtration through a 250 μm filter and centrifugation at 1000 g for 10 min. Finally, the pellet was resuspended in a cultural medium containing DMEM (Sigma-Aldrich, St. Louis, MO, USA), 10% fetal bovine serum (FBS; Thermo Fisher Scientific, Waltham, MA, USA), 4 mM *L*-glutamine, 4 nM insulin, 0.004% gentamicin, and 25 μg/mL sodium ascorbate (Sigma-Aldrich, St. Louis, MO, USA). The obtained suspension of preadipocytes were placed on round coverglasses (25 mm in diameter), which were then put in 35 mm Petri dishes. On the ninth day of culture, the cells form a monolayer and become differentiated.

### 4.2. The Measurement of Cytosolic Calcium Concentration ([Ca^2+^]_i_)

The measurement of [Ca^2+^]_i_ was performed by fluorescent microscopy using Fura-2/am (Molecular probes, USA), a ratiometric fluorescent calcium indicator as described in detail previously [47]. Cells were loaded with the probe dissolved in Hanks balanced salt solution (HBSS), containing 10 mM HEPES and 200 µM L-arginine, pH 7.4, at a final concentration of 5 μM at 37 °C for 40 min with subsequent 15 min washout. We used an Axiovert 200M based imaging system (Carl Zeiss, Berlin, Germany) equipped with a HBO100 mercury lamp, AxioCam HSm CCD camera, and MAC5000 high-speed excitation filter wheel. Fura-2 fluorescence was excited at two wavelengths using band-pass filters BP 340/30 and BP 387/15; fluorescence was registered in the wavelength range of 465–555 nm. Excitation light intensity was lowered using 25% and 5% neutral density filters in order to prevent phototoxicity. Image frames were acquired at 3 s intervals. The time lapse image sequences were analyzed using ImageJ 1.44 (NIH Image, Bethesda, MD, USA).

### 4.3. Immunocytochemical Method

In order to detect RyR and IP_3_R in cells, we used an immunocytochemical assay. The cells were fixed with 4% paraformaldehyde + 0.25% glutaraldehyde in PBS for 20 min and washed three times with ice-cold PBS for 5 min. Glutaraldehyde was added into the fixative solution to minimize washing of BDNF from cells during permeabilization. To permeabilize cells, we used 0.1% Triton X-100 solution for 15 min. Fixed cells were incubated in 10% donkey serum for 30 min at room temperature to block non-specific antibody binding sites. The cells were then incubated with primary antibodies against investigated proteins for 12 h at 4 °C. The fixed cells were subsequently washed with PBS (3 times for 5 min) and probed with secondary antibodies conjugated with fluorescent label manual. We used purified mouse monoclonal anti-ryanodine receptor antibody (Abcam, Cambridge, UK, ab2868), rabbit polyclonal antibody to IP3 (inositol 1,4,5-trisphosphate)-receptor (Sigma-Aldrich, St. Louis, MO, USA, 07-1210), goat anti-mouse IgG (H+L), highly cross-adsorbed secondary antibody (Alexa Fluor-633) (Thermo Fisher Scientific, Waltham, MA, USA, Cat. No. A-21052), and donkey polyclonal secondary antibody to rabbit IgG-H&L (Alexa Fluor-555) (Abcam, Cambridge, UK, RRID: AB_2636997). The fluorescence of antibodies was visualized with an inverted confocal microscope Leica TCS SP5 (Leica, Hamburg, Germany).

### 4.4. Animal Model of Obesity and Type 2 Diabetes

The animal model of obesity and type 2 diabetes (T2D), described previously for rats [71], was used in present experiments. White mice were housed in a 21 ± 1 °C controlled room under a 12 h light–dark cycle. Animals had free access to food and water and were housed with 5 mice per cage. To induce various stages of obesity, we used a 5-to-8-month course of high-fat diet (HFD) feeding, based on the addition of the lard (200–400 mg/day/animal) to the standard chow of rodents, beginning the experiments with 7–8-week-old mice. Like the model of obesity presented in [5], this model is heterogeneous in terms of weight gain, liver histological scoring, blood pressure, fasting glucose, etc. Up to 8% lf mice kept on HFD exhibited lipodystrophy with initial minimal weight gain followed by weight loss, while 7–8% of obese animals suffered from liver cancer, and 4–5% from abdominal ascites. The mice that gained weight more rapidly (HFD responders, 30% of group) were engaged in the experiments.

Here, obese 6–7-month-old fat-responsive mice were characterized in a fasted state (12–14 h) by elevated levels of blood glucose (8.01 ± 0.17 vs. 6.4 ± 0.15 mM, SD), insulin (1.95 ± 0.11 vs. 0.44 ± 0.16 ng/mL, SD), and total cholesterol (3.5 ± 0.13 vs. 2.35 ± 0.09 mM, SD), raised blood pressure (BP = 143 ± 71.5 vs. 121 ± 2.4 mm Hg, SD), and macromolecular liver steatosis. The animals with T2D (≥10 month) displayed further increase of the concentrations of glucose (11.9 ± 0.41 mM), insulin (3.39 ± 0.13 ng/mL), and cholesterol (4.33 ± 0.18 mM) concentrations, the rise of BP (177 ± 3 mm Hg), and the occurrence of varied stages liver fibrosis. Total body mass, arterial glucose, and blood pressure (BP) were measured once a month. Tail BP was registered as tail cuff pressure with BP-2000, USA/Visitech System.

### 4.5. Metabolite and Blood Parameters Determinations

Mice were killed by CO_2_ narcosis/cervical dislocation after a fasting period of 12–14 h. Tail venous blood glucose was measured using Glucometer Elite (Bayer, Elkhart, IN, USA). Mixed blood was collected in heparinized tubes and processed by centrifugation, and blood plasma was used to measure insulin (with Rat/Mouse Insulin ELISA|EZRMI-13K-Merck Millipore), total cholesterol, triglycerides, and free fatty acids (using appropriate enzymatic Kits (Diacon, Pushchino, Moscow Oblast, Russia). All measured blood parameters are presented in Table 2.

### 4.6. Statistical Analysis

All presented data were obtained from at least three cell cultures from two to three different passages. n—number of the experiments. All values are given as mean ± standard error (SE). The differences between the columns were estimated with paired *t*-test. The statistical tests were performed with GraphPad Prism 5 software (San Diego, CA, USA).

## 5. Conclusions

Considering NOS-G as the system that may integrate hormonal signals involved in the control of NO bioavailability, we may conclude that the application of ACh, ANP, insulin, NO donors, PDE inhibitors, etc., might be ineffective to raise PKG1 and NOS-G activity in the cells of diabetic animals with deregulated Ca^2+^ signaling pathways, such as the cells of “sick” fat depots, pancreatic and vascular cells, and interstitial cells of gastrointestinal tract, etc.

Apparently, the deregulation of Ca^2+^ and second messenger signaling systems represent a gradual tissue specific process, characterized by progressive loss of feedback control and signaling functions, depending on the stage of obesity development, environmental factors, etc. It is obvious that modern treatment modalities should take into account the alterations in complex feedback control of such systems.

## Figures and Tables

**Table 1:** Contribution of PLC-G and NOS-G into rhythmic activity evoked by various hormones in cultured adipocytes (9DIV) of eWAT.

	Agonists and Their Concentrations						
	ACh 1–5 µM	NE 1–5 µM	ANP 1–10 µM	CCK 3–20 nM	ANGII 0.2– 1 µM	BK 0.3–10 µM	Ins 3–20 nM
**Receptors and proteins**	**m3** **Gβγ**	**α1** **Gαq**	**NPR-A** **Gα**	**CCK-B** **Gαq**, **Gβγ**	**AT-1** **Gαq**, **Gβγ**	**B2R** **Gαq**, **Gβγ**	**RTK** **TK**, **cSrc**
**PLC-G,** **% of cells with rhythmic activity**	**–**	**30–40**	**–**	**25–40**	**25–40**	**30–40**	**20–30**
**NOS-G,** **% of cells with rhythmic activity**	**70–80**	**–**	**30–40**	**20–40**	**30–35**	**25–30**	**15–25**
**Periods of Ca^2+^-oscillations (s)**	5–60 100–300	20–75 100–300	20–50 200–300	25–30 300–500	20–50 75–200	10–30 200–500	20–30 50–150

**Table 2:** Characteristics of control, obese, and diabetic mice, including age, weight, blood pressure, blood glucose, insulin, and lipids profile. Control—2-month-old mice. Obese 7-month-old mice, after 5 months of high-fat feeding. Ten-month-old diabetic mice (T2D), after 8 months of high-fat feeding. Cholest—total cholesterol. TG—triglycerides. FFA—total free fatty acids. All details are given in the text of Methods. All data are presented as mean ± SE.

Age (Month)	Weight (g)	Glucose (mM)	Insulin (ng/mL)	Cholest (mM)	TG (mM)	FFA (mM)	Blood Pressure (mmHg)
2	24.4 ± 0.56	6.4 ± 0.15	0.51 ± 0.04	2.35 ± 0.09	1.48 ± 0.08	1.14 ± 0.11	121 ± 2.4
7	42.5 ± 0.72	8.01 ± 0.21	1.95 ± 0.11	3.5 ± 0.13	1.92 ± 0.15	1.82 ± 0.12	143 ± 1.53
10	56.4 ± 1.42	11.9 ± 0.41	3.39 ± 0.13	4.33 ± 0.18	2.62 ± 0.21	1.84 ± 0.16	177 ± 2.96

## Abbreviations

[Ca^2+^]_i_	intracellular free calcium concentration
PKB	protein kinase B
PI3Kα, γ	lipid kinases α, γ
eNOS	endothelial NO-synthase
AMPKα	AMP-activated protein kinase α
ACh	acetylcholine
NE	norepinephrine
BK	bradykinin
CCK	cholecystokinin
AngII	Angiotensin II
Ins	Insulin
Gβγ	G-protein βγ
G_αq_	G-protein _αq_
α1,2-*AR*	α1,2-adrenoreceptors
β1-3-*AR*	β1-3-adrenoreceptors
eWAT	epididymal white adipose tissue
CICR	calcium- induced calcium release
NFL	negative feedback loop
PFL	positive feedback loop
*m3-MR*	*m3*-muscarinic receptors
ANP	atrial natriuretic peptide
NOS-G	eNOS- dependent rhythmic generator
PLC-G	PLC- dependent rhythmic generator
Gαs	G-protein αs
GPCR	G-protein coupled receptors
RTK	tyrosine kinase coupled receptors
T2D	type 2 diabetes
VP	vasopressin
ANP	atrial natriuretic peptide
ET-1	endothelin-1
PE	phenylephrine
SERCA	Ca^2+^-ATPase of endoplasmic reticulum
PMCA	Ca^2+^-ATPase of plasmalemma
AC	adenylate cyclase
PKA	protein kinase A
VEGF	vascular endothelial growth factor
CCK	cholecystokinin
BA	Bile acids
IP3R1,2	inositol 1,4,5-trisphosphate receptors subtypes 1,2
CaMKIIβ	Ca^2+^/calmodulin-dependent protein kinase IIβ
CaMKK	Ca^2+^/calmodulin-dependent kinase kinase
IP3	inositol 1,4,5-trisphosphate
PLCβ,γ	phospholipase C β,γ
sGC	soluble guanylate cyclase
PKG1	cGMP-dependent protein kinase 1
CD38	ectoenzyme ADP-ribosyl cyclase
PKB	protein kinase B
ARC	ADP-ribosyl cyclase
cADPr	cyclic ADP-ribose
TK	membrane tyrosine kinase
cSrc	cytosolic tyrosine kinase
RyR2,3	ryanodine receptors subtypes 2,3
RGS	Regulator of G protein signaling
IRAG	IP3R-associiated protein kinase G substrate
